# Advanced cueing of auditory stimulus to the head induces body sway in the direction opposite to the stimulus site during quiet stance in male participants

**DOI:** 10.3389/fnhum.2022.1028700

**Published:** 2022-12-08

**Authors:** Naoki Hamada, Hiroshi Kunimura, Masakazu Matsuoka, Hitoshi Oda, Koichi Hiraoka

**Affiliations:** ^1^Department of Rehabilitation Science, School of Medicine, Osaka Metropolitan University, Habikino, Japan; ^2^Graduate School of Comprehensive Rehabilitation, Osaka Prefecture University, Habikino, Japan

**Keywords:** defense mechanism, postural control, auditory stimulus, tactile stimulus, center of pressure, electromyography

## Abstract

Under certain conditions, a tactile stimulus to the head induces the movement of the head away from the stimulus, and this is thought to be caused by a defense mechanism. In this study, we tested our hypothesis that predicting the stimulus site of the head in a quiet stance activates the defense mechanism, causing a body to sway to keep the head away from the stimulus. Fourteen healthy male participants aged 31.2 ± 6.8 years participated in this study. A visual cue predicting the forthcoming stimulus site (forehead, left side of the head, right side of the head, or back of the head) was given. Four seconds after this cue, an auditory or electrical tactile stimulus was given at the site predicted by the cue. The cue predicting the tactile stimulus site of the head did not induce a body sway. The cue predicting the auditory stimulus to the back of the head induced a forward body sway, and the cue predicting the stimulus to the forehead induced a backward body sway. The cue predicting the auditory stimulus to the left side of the head induced a rightward body sway, and the cue predicting the stimulus to the right side of the head induced a leftward body sway. These findings support our hypothesis that predicting the auditory stimulus site of the head induces a body sway in a quiet stance to keep the head away from the stimulus. The right gastrocnemius muscle contributes to the control of the body sway in the anterior–posterior axis related to this defense mechanism.

## Introduction

We frequently observed the head of the participants moving forward in a quiet stance when a coil, attached to a magnetic stimulator, was placed over the back of the head for transcranial magnetic stimulation (TMS). The participants could have predicted the forthcoming TMS when the coil was placed over the back of the head. Predicting the perturbation changed the motor response to the perturbation (Caudron et al., 2008; Jacobs et al., 2008; Matsuoka et al., 2020) and influenced the vestibular function when the participants were in the stance (Guerraz and Day, 2005). Previous findings are consistent with the hypothesis that postural control in a quiet stance is influenced by predicting the forthcoming stimulus. Based on this view, a forward sway of the body induced by placing the coil at the back of the head is likely due to predicting forthcoming TMS at this site. When TMS is given, the coil produces a clicking sound. The clicking sound of TMS elicits a large auditory-evoked potential, indicating that the clicking sound activates the auditory cognitive process (Nikouline et al., 1999; Tiitinen et al., 1999). Accordingly, the forward sway of the head induced by placing the TMS coil over the back of the head may be explained by a view that humans move the head forward due to the prediction of the clicking sound.

This event, the forward sway of the body induced by the prediction of the clicking sound over the back of the head, may be explained by a defense mechanism. Mammalians move their body to keep them away from physical offense (Graziano and Cooke, 2006). The startle response induced by a loud sound and the withdrawal reflex induced by the noxious tactile stimulus are typical examples of the defense response (Sherrington, 1910; Landis and Hunt, 1939; Pfeiffer, 1962; Koch, 1999; Yeomans et al., 2002; Graziano and Cooke, 2006; Davis et al., 2009; Jure, 2020). These defense responses have been thought to play a role in constructing safety margins around the body for keeping the body away from the noxious stimulus.

Mammalians, including humans, respond to the stimulus given within defensive peripersonal space (flight zone), defined as the space directly surrounding the body at a grasping distance (Rizzolatti et al., 1997; Vagnoni and Longo, 2019; Rabellino et al., 2020). The cortically mediated excitatory long-loop reflex was facilitated when the stimulated hand was within the defensive peripersonal space (Versace et al., 2019). The blink reflex was found to be facilitated when a stimulated hand (electrical stimulation to the median nerve at the wrist) was close to the face, called the hand–blink reflex in humans (Sambo et al., 2012; Biggio et al., 2019). Those findings indicate that the defense mechanism is activated particularly when the tactile stimulus is close to the head. When tactile stimulation (air puff) was given to the head, the monkeys moved their heads away from the stimulus (Cooke et al., 2003). This finding indicates that tactile stimulus to the head activates the defense mechanism to keep the body away from the stimulus. Postural control is sensitive to the motion of the audible environment (Stoffregen et al., 2009). There is auditory peripersonal space around the head in humans (Ladavas et al., 2001). Those findings indicate that the defense mechanism is likely activated not only by a tactile stimulus but also by an auditory stimulus. Based on this view, we hypothesized that predicting the stimulus site of the head induces a body sway to keep the head away from the stimulus mediated by the defense mechanism (Hypothesis 1). We tested this hypothesis by investigating the effect of predicting forthcoming auditory or tactile stimulus sites of the head-on-body sway in a quiet stance.

In a quiet stance, ankle muscles control the body's sway (Winter et al., 1998; Warnica et al., 2014). Accordingly, the body sway induced by predicting the stimulus site must be associated with a change in ankle muscle activity. More specifically, anterior–posterior body sway is mainly controlled by the ankles (Winter, 1993). Based on this finding, the ankle muscles likely respond to the prediction of the stimulus site particularly in the sagittal plane (forehead or back of the head), if the deviation of the body is mediated by the defense mechanism (Hypothesis 2). This hypothesis was also examined in the present study.

The amount of body sway also reflects the postural control in a quiet stance. A static sound cue was found to decrease the amount of body sway in stance (Gandemer et al., 2017). A rotating sound cue was found to decrease the amount of body sway in stance (Gandemer et al., 2014). Auditory stimulus reduced the amount of body sway in stance (Agaeva and Altman, 2005; Ross and Balasubramaniam, 2015; Ross et al., 2016). Predicting the sound stimulus may activate the mechanism that is the same as the mechanism underlying those previous findings. When humans maintained their stance on an elevated ground surface, the COP displacement decreased, indicating that postural threat decreases body sway in a quiet stance (Carpenter et al., 2001; Brown et al., 2006). These previous findings are explained by the view that emotional stress decreases the amount of body sway in a stance. Predicting the stimulus to the head likely induces emotional stress, and thus, may decrease the amount of the body sway. Based on those, we hypothesized that predicting the sound and/or tactile stimulus decreases the amount of body sway in a quiet stance (Hypothesis 3).

## Methods

### Participants

A total of 14 healthy male participants aged 31.2 ± 6.8 years participated in this study. There are gender differences in physical characteristics, motor performance (Hamill et al., 1977; Thomas and French, 1985), and postural control (Gribble et al., 2009). The inter-individual variability of measures representing postural control in female participants is greater than that in male participants (Kahraman et al., 2018). Based on those previous findings, to exclude the variability of postural responses caused by gender differences, and to minimize the inter-individual variability of postural control, only male participants were recruited. All the participants were right-footed according to the revised version of the Waterloo Footedness Questionnaire (Elias et al., 1998; Zverev, 2006). The participants did not have a history of orthopedic or neurological disorders. The experimental protocol was explained, and the participants gave their written informed consent to participate in this experiment. All the procedures were approved by the Ethics Committee of Osaka Prefecture University.

### Apparatus

A gravicorder was used to measure the center of pressure (COP) (Static Sensograph, 1G06, NEC Sanei, Tokyo). A speaker producing sound with 45 dB of sound pressure was placed around the head for the trial block investigating the effect of the predicted auditory stimulus site of the head (auditory stimulus trial block). Electrical stimulus electrodes, providing an electrical tactile stimulus, were placed over the skin at the forehead (5 cm caudal to the nasion), inion, or the left or right mastoid in the tactile stimulus trial block. The loci of the nasion, inion, and mastoids were determined via palpation over the skin. The distance between the two electrodes placed at each site was 1 cm. A display showing pictures predicting the presence and site of the stimulus (S-12140, Takei kiki, Tokyo, Japan) was placed 1 m in front of the participants. The participants wore liquid crystal goggles (T.K.K.2275, Takei Kiki, Tokyo, Japan). The goggles were either opaque for visual occlusion or transparent to allow vision. Electrodes recording the electromyographic (EMG) activity in the left gastrocnemius medius (GM), right GM, left tibialis anterior (TA), and right TA muscles were placed over the belly of the muscles. The inter-electrode distance in each muscle was 2 cm. The EMG signals were amplified with a pass-band filter from 15 Hz to 1 kHz using amplifiers (MEG 5200, Nihon Kohden, Tokyo). Analog signals from the gravicorder and EMG amplifiers were digitized using an A/D converter (PowerLab/8SP and 2sp; ADInstruments, Colorado Spring, CO, USA) at a sampling rate of 2 kHz, and the digitized signals were stored in a personal computer.

### Procedure

**Figure 1:**
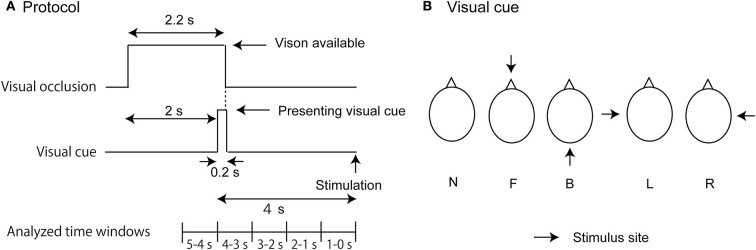
Experimental design. The time protocol in each trial is shown in **(A)**. Visual cues indicating a bird's-eye view of the stimulus site are shown in **(B)**. In **(B)**, each abbreviation indicates the stimulus condition: L, L condition (cue predicting the stimulus to the left side of the head); R, R condition (cue predicting the stimulus to the right side of the head); F, F condition (cue predicting the stimulus to the forehead); and B, B condition (cue predicting the stimulus to the back of the head).

The participants maintained the stance with the feet together over the gravicorder. Before beginning each trial, the liquid crystal goggles were opaque so that their vision was occluded. An experimenter monitored the COP and initiated the trial when the COP was stable. The goggles became transparent in the time window between 0 and 2.2 s after the beginning of the trial (Figure 1A). The goggles were opaque after this time until the time at which the stimulus was given so that the influence of the visual information other than the visual cue was minimum. A picture depicting a bird's-eye view of the head was presented on the display in the time window between 2 and 2.2 s after the beginning of the trial. In this picture, an arrow predicting the stimulus site was given in the trials with the stimulation (Figure 1B). The bird's-eye view of the head was presented, but the arrow was not presented in the trials without the stimulation (N condition). The meaning of this arrow was explained to the participants before beginning the experiment. The goggles were again opaque after this moment until the end of the trial.

An auditory stimulus was given 4 s after the onset of the visual cue in the auditory stimulus trial block (Figure 1A). The auditory stimulus was given with a speaker placed at the site, where the arrow in the picture of the visual cue was predicted. The speaker was 5 cm away from the stimulus site of the head. An electrical tactile stimulus was given 4 s after the onset of the visual cue in the tactile stimulus trial block. In the tactile stimulus trial block, an electrical stimulus was given. The intensity of the stimulus was 1.5 times the intensity of the tactile perceptual threshold. The stimulus intensity increased from the subthreshold to the suprathreshold level in an increment of 0.47 V every 1 s, and the least intensity at which the participants perceived the stimulus was considered to be the stimulus intensity at the tactile perceptual threshold. The tactile stimulus trial block was conducted 20 min after the auditory stimulus trial block. The stimulus was given at one of the four sites (forehead [F], back of the head [B], or left [L] or right side of the head [R]). Each trial lasted 6 s. The stimulus at each site was given in 10 trials. No stimulus was given in the 10 trials of the N condition. Thus, 50 trials were conducted in each trial block. One of these five conditions was randomly assigned in each trial.

### Data analysis

The COP in the medial–lateral axis was called COPx, and that in the anterior–posterior axis was called COPy. The mean COP position was calculated at each 1 s. The positive value of the COPx position indicates the rightward deviation of the COP, and that of the COPy position indicates the forward deviation of the COP. The mean and standard deviation of the COP were calculated at every 1 s (5–4, 4–3, 3–2, 2–1, and 1–0 s before the stimulus onset). The standard deviation of the COP represented the amount of the body sway at each 1 s. The EMG trace in each trial was rectified, and the amplitude of the rectified EMG trace was averaged at each 1 s.

A repeated-measures two-way ANOVA was conducted to test the main effects on all measures: time (five levels; 5–4, 4–3, 3–2, 2–1, and 1–0 s before the stimulus onset) and stimulation (five levels; N, F, B, L, and R conditions). The Greenhouse–Geisser correction was conducted on the results whenever Mauchly's test of sphericity was significant. If there was a significant interaction between the main effects, then a test of the simple main effect was conducted. If there was a significant main effect or simple main effect, then a multiple comparison test (Bonferroni's test) followed it. The alpha level was 0.05. Excel Toukei 2010 ver. 1.13 (Social Survey Research Information, Tokyo) was used for the statistical analysis. The data in the results were expressed as the mean and standard error of the mean. The data, normalized by subtracting the average value at 1–0 s before the onset of the visual cue (5–4 s before the stimulus) from the values averaged at every 1 s in the time window between 4 and 0 s before the stimulus, were used to depict the mean and error bars of the figures.

## Results

### COP position

#### Auditory stimulus trial block

**Figure 2:**
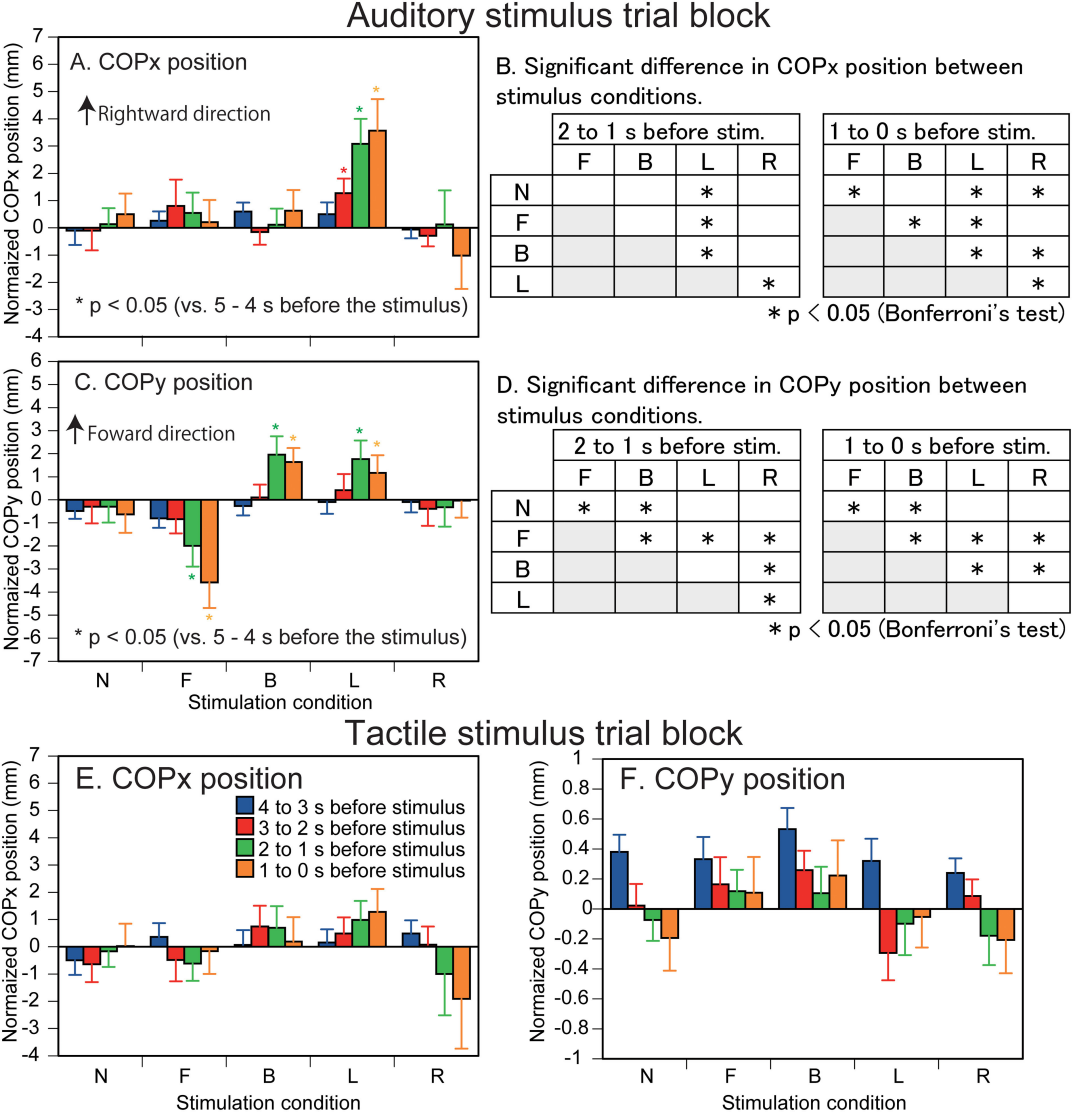
Effect of predicting the auditory **(A–D)** and tactile stimulus site **(E,F)** on the COP position. Bars indicate the mean, and error bars indicate standard errors of the mean **(A,C,E,F)**. The data are normalized by subtracting the average value at 1–0 s before the onset of the visual cue (5–4 s before the stimulus) from the values averaged at every 1 s in the time window between 4 and 0 s before the stimulus. Asterisks indicate significant differences in the COP position compared with the COP position at the time 5–4 s before the stimulus (1–0 s before the visual cue) in the same condition. Results of Bonferroni's test examining the difference between the stimulus conditions are shown in **B** and **D**. N, trials with the cue predicting non-stimulation; F, trials with the cue predicting the stimulus to the forehead; B, trials with the cue predicting the stimulus to the back of the head; L, trials with the cue predicting the stimulus to the left side of the head; R, trials with the cue predicting the stimulus to the right side of the head.

**Figure 3:**
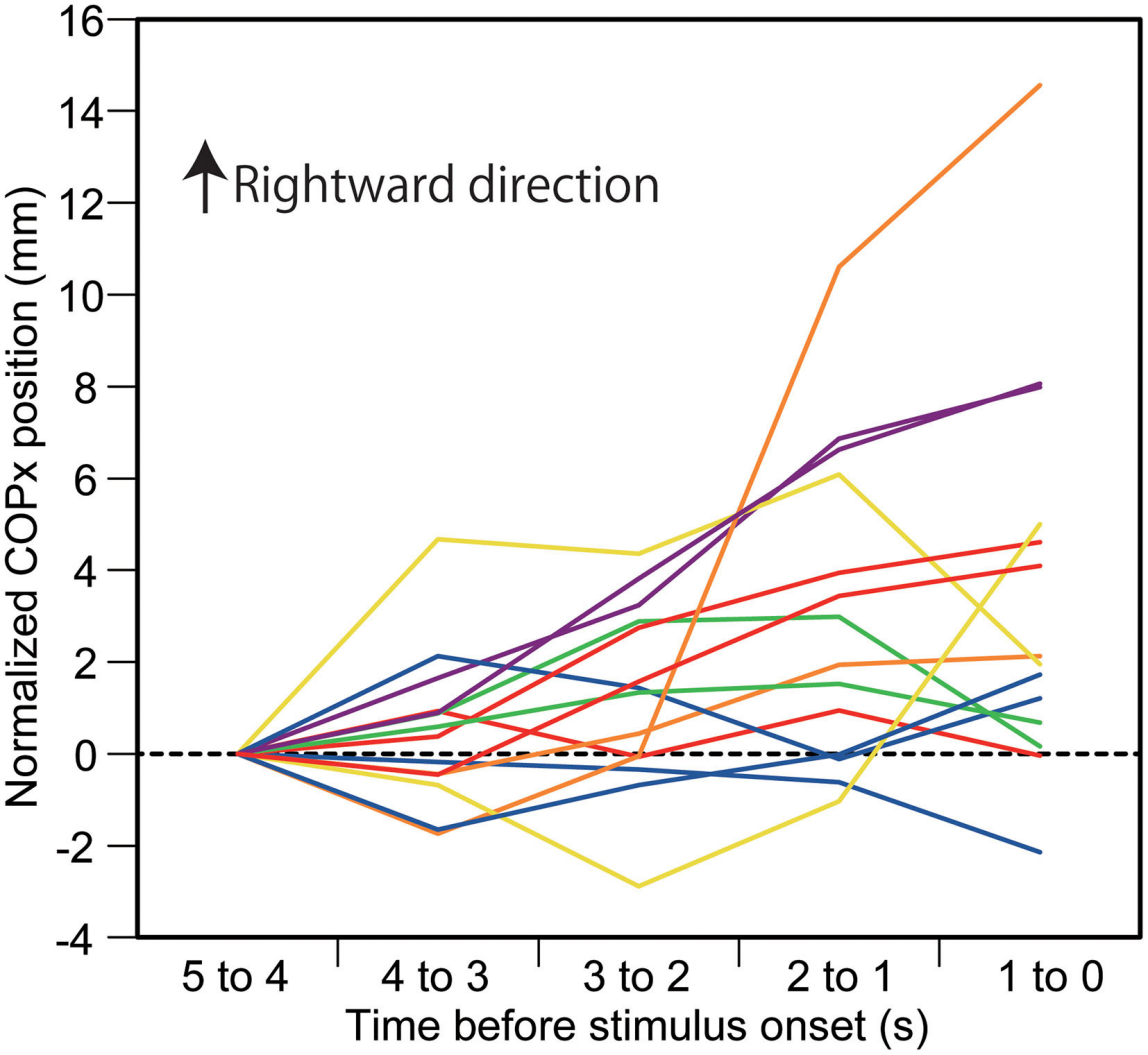
Individual data on the COPx position in auditory stimulus trial block. Each line indicates the COPx position in each participant. The data are normalized by subtracting the average value at 1–0 s before the onset of the visual cue (5–4 s before the stimulus) from the values averaged at every 1 s in the time window between 4 and 0 s before the stimulus.

The effect of predicting the auditory stimulus site on the COPx position is shown in Figure 2A. There was a significant interaction between the main effects [*F*_(16,208)_ = 2.338, *p* = 0.003]. The test of the simple main effect revealed a significant effect of time in the L condition [*F*_[4,260]_ = 8.172, *p* < 0.001]. The COPx position significantly deviated to the rightward direction at 3–0 s before the stimulus compared with the 5–4 s before the stimulus (1–0 s before the visual cue) in the L condition (*p* < 0.05). The COPx position significantly deviated to the rightward direction at 2–0 s before the stimulus compared with the 4–3 s before the stimulus in the L condition (*p* < 0.05). The COPx position significantly deviated to the rightward direction at 2–0 s before the stimulus compared with the 3–2 s before the stimulus in the L condition (*p* < 0.05). The test of the simple main effect revealed a significant effect of the stimulus at 2–1 s [*F*_[4,260]_ = 3.271, *p* = 0.012] and at 1–0 s [*F*_[4,260]_ = 6.553, *p* < 0.001] before the stimulus. As shown in Figures 2A,B, the COPx position was significantly deviated rightward in the L condition compared with the N condition at 2–0 s before the stimulus but deviated leftward compared with the N condition in the R condition at 1–0 s before the stimulus (*p* < 0.05). The COPx position significantly deviated rightward in the F condition compared with the N condition at 1–0 s before the stimulus (*p* < 0.05). The COPx position in the L condition for individual participants is shown in Figure 3.

The effect of predicting the auditory stimulus site on the COPy position is shown in Figure 2C. There was a significant interaction between the main effects [*F*_(4.727,61.451)_ = 5.611, *p* < 0.001]. The test of the simple main effect revealed a significant effect of time in the B [*F*_(4,260)_ = 4.522, *p* = 0.002], F [*F*_(4,260)_ = 8.193, *p* < 0.001], and L conditions [*F*_(4,260)_ = 2.711, *p* = 0.031]. The COPy position deviated backward at 2–0 s before the stimulus compared with that at 5–2 s before the stimulus and at 1–0 s before the stimulus compared with that at 2–1 s before the stimulus in the F condition (*p* < 0.05). The COPy position deviated forward at 2–0 s before the stimulus compared with that at 5–2 s before the stimulus in the B condition (*p* < 0.05). The COPy position deviated forward at 2–1 s before the stimulus compared with that at 5–2 s before the stimulus, and at 1–0 s before the stimulus compared with that at 5–3 s before the stimulus in the L condition (*p* < 0.05). The test of the simple main effect revealed a significant simple main effect of the stimulus at 2–1 s [*F*_(4,260)_ = 4.264, *p* = 0.002] and at 1–0 s [*F*_(4,260)_ = 8.043, *p* < 0.001] before the stimulus. As shown in Figure 2D, the COPy position in the F condition significantly deviated backward compared with the N condition 2–0 s before the stimulus (*p* < 0.05). The COPy position in the B condition significantly deviated forward compared with the N condition 2–0 s before the stimulus (*p* < 0.05).

#### Tactile stimulus trial block

The COPx position is shown in Figure 2E. There was neither a significant main effect of time [*F*_(1.905,24.77)_ = 0.058, *p* = 0.937] nor stimulus [*F*_[2.129,27.675]_ = 0.442, *p* = 0.660] without significant interaction between the main effects [*F*_(16,208)_ = 1.409, *p* = 0.140]. The COPy position is shown in Figure 2F. There was neither a significant main effect of time [*F*_(2.207,28.691)_ = 1.790, *p* = 0.182] nor stimulus [*F*_(4,52)_ = 2.445, *p* = 0.058] without significant interaction between the main effects [*F*_(3.404,44.250)_ = 1.652, *p* = 0.186].

### COP displacement

#### Auditory stimulus trial block

**Figure 4:**
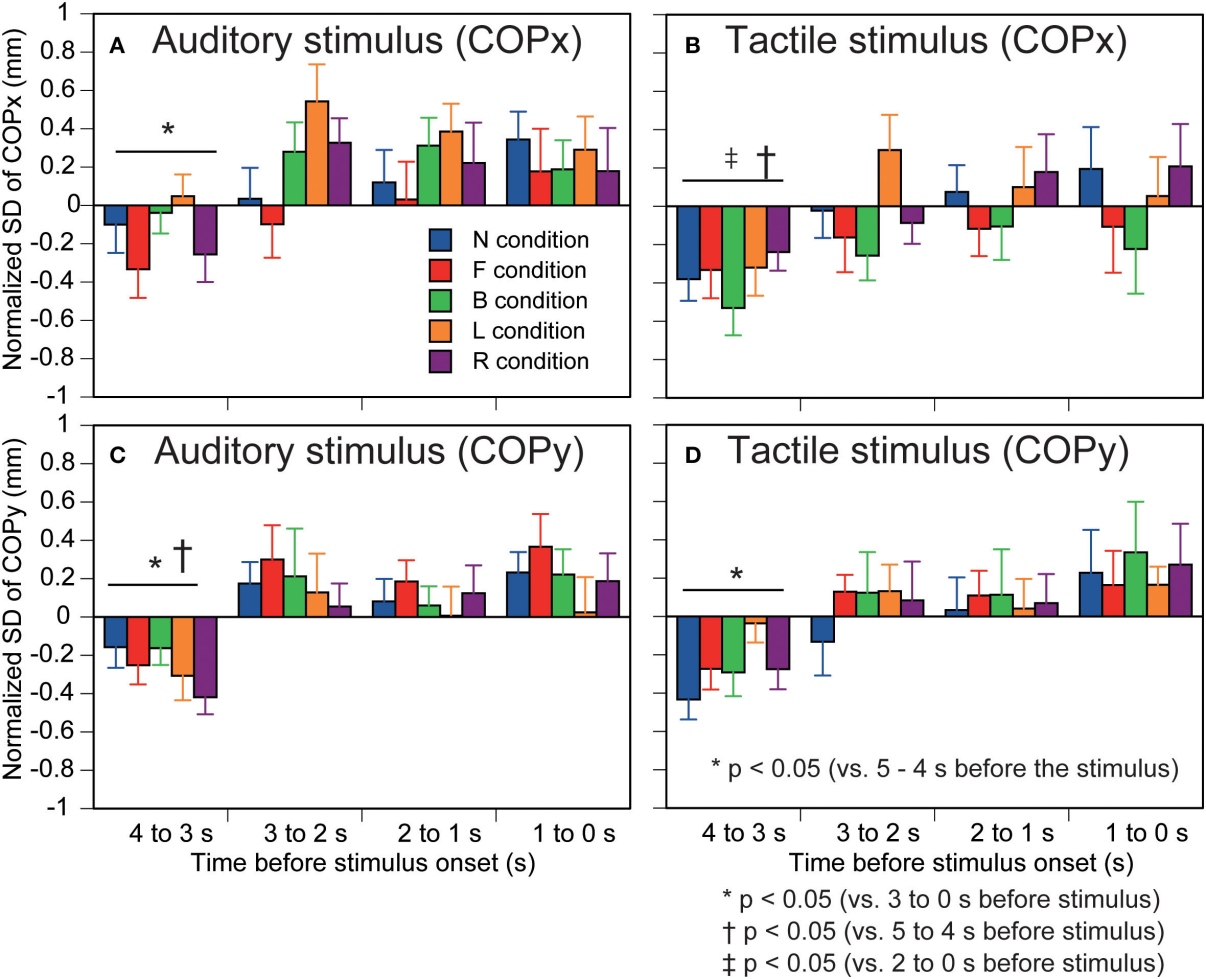
Effect of predicting the auditory or tactile stimulus on the standard deviation of COP. Auditory stimulus trial blocks are in the left panels **(A,C)** and tactile stimulus trial blocks are in the right panels **(B,D)**. The upper panels are COPx **(A,B)** and lower panels are COPy **(C,D)**. Bars indicate the mean, and error bars indicate standard errors of the mean. The data are normalized by subtracting the average value at 1–0 s before the onset of the visual cue (5–4 s before the stimulus) from the values averaged at each 1 s in the time window between 4 and 0 s before the stimulus. N, trials with the cue predicting non-stimulation; F, trials with the cue predicting the stimulus to the forehead; B, trials with the cue predicting the stimulus to the back of the head; L, trials with the cue predicting the stimulus to the left side of the head; R, trials with the cue predicting the stimulus to the right side of the head. Effect of predicting the auditory or tactile stimulus on the standard deviation of COP. Auditory stimulus trial blocks are in the left panels **(A,C)** and tactile stimulus trial blocks are in the right panels **(B,D)**. The upper panels are COPx **(A,B)** and lower panels are COPy **(C,D)**. Bars indicate the mean, and error bars indicate standard errors of the mean. The data are normalized by subtracting the average value at 1–0 s before the onset of the visual cue (5–4 s before the stimulus) from the values averaged at each 1 s in the time window between 4 and 0 s before the stimulus. N, trials with the cue predicting non-stimulation; F, trials with the cue predicting the stimulus to the forehead; B, trials with the cue predicting the stimulus to the back of the head; L, trials with the cue predicting the stimulus to the left side of the head; R, trials with the cue predicting the stimulus to the right side of the head.

The standard deviation of the COPx is shown in Figure 4A. There was no significant interaction between the time and stimulus [*F*_(16,208)_ = 1.301, *p* = 0.199]. There was a significant effect of the stimulus [*F*_(2.348,30.523)_ = 3.536, *p* = 0.035]. The standard deviation of the COPx in the R condition was significantly greater than that in the B condition (*p* < 0.05). There was a significant effect of the time [*F*_(1.823,23.694)_ = 5.530, *p* = 0.012]. The standard deviation of the COPx at 4–3 s before the stimulus was significantly smaller than that at 3–0 s before the stimulus (*p* < 0.05).

The standard deviation of the COPy is shown in Figure 4C. There was no significant interaction between the main effects [*F*_(16,208)_ = 0.566, *p* = 0.907]. There was no significant effect of the stimulus [*F*_(4,52)_ = 1.456, *p* = 0.229]. There was a significant main effect of time [*F*_(2.049,26.634)_ = 11.515, *p* < 0.001]. The standard deviation of the COPx at 4–3 s before the stimulus was significantly smaller than that at the other times (*p* < 0.05).

#### Tactile stimulus trial block

The standard deviation of the COPx is shown in Figure 4B. There was no significant interaction between the time and stimulus [*F*_(16,208)_ = 0.999, *p* = 0.459]. There was a significant main effect of time [*F*_(1.856,24.133)_ = 4.215, *p* = 0.029]. The standard deviation of the COPx at 4–3 s before the stimulus was significantly smaller than that at 5–4 and 2–0 s before the stimulus (*p* < 0.05). There was no significant main effect of the stimulus [*F*_(2.021,26.267)_ = 1.006, *p* = 0.380].

The standard deviation of the COPy is shown in Figure 4D. There was no significant interaction between the time and stimulus regarding the standard deviation [*F*_(16,208)_ = 0.643, *p* = 0.847]. There was a significant main effect of time [*F*_(2.156,28.032)_ = 7.237, *p* = 0.002]. The standard deviation of the COPx at 4–3 s before the stimulus was significantly smaller than that at 3–0 s before the stimulus (*p* < 0.05). There was no significant main effect of the stimulus [*F*_(4,52)_ = 2.266, *p* = 0.075].

### EMG

#### Muscles with a significant interaction between the main effects

**Figure 5:**
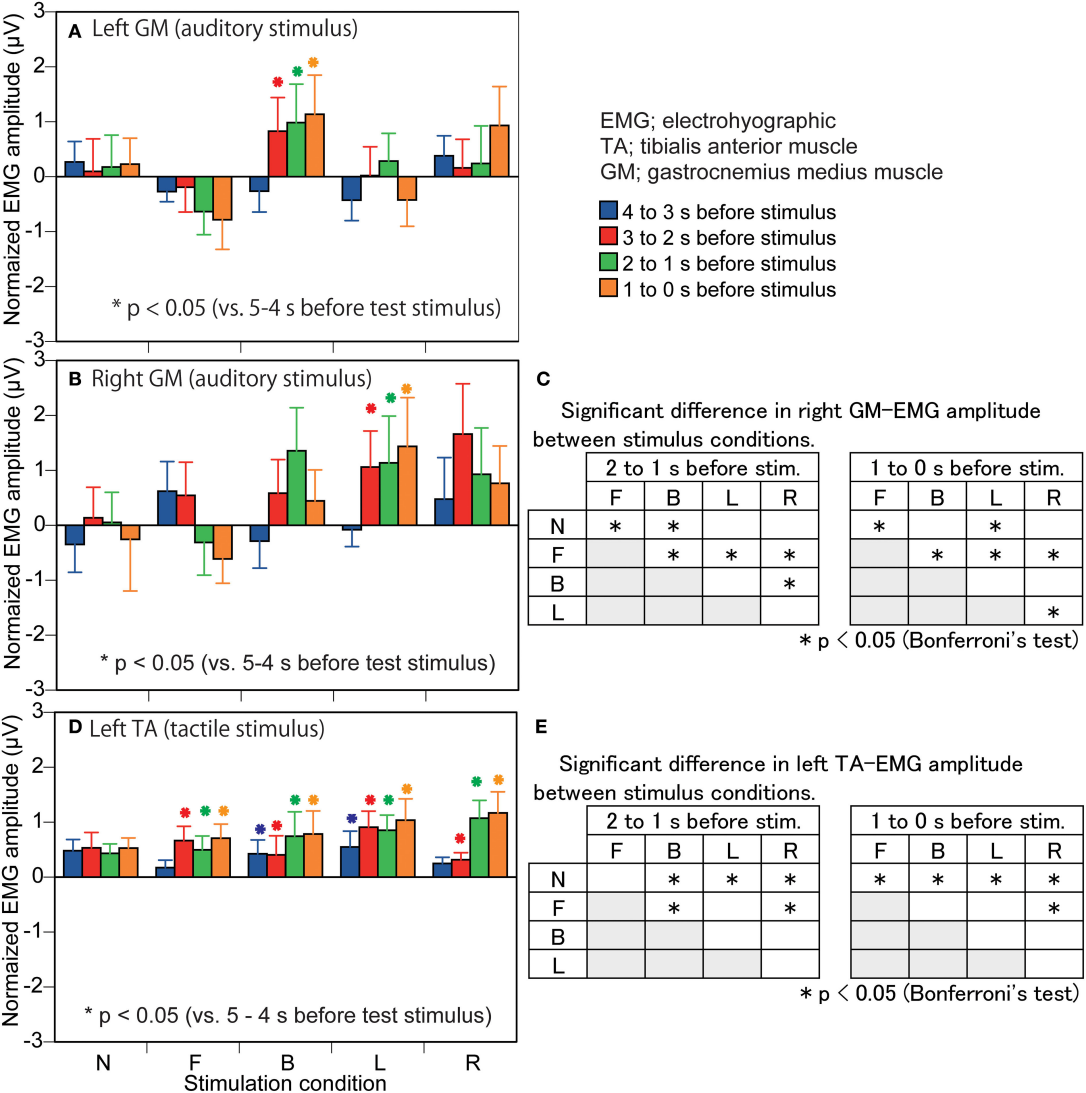
Effect of predicting the stimulus site on the EMG amplitude in the muscles in which a significant interaction between the main effects is revealed. The data are normalized by subtracting the average value at 1–0 s before the onset of the visual cue (5–4 s before the stimulus) from the values averaged at each 1 s in the time window between 4 and 0 s before the stimulus. Bars indicate the mean, and error bars indicate standard errors of the mean in the left panels **(A,B,D)**. Asterisks indicate significant differences from mean amplitude at the time 5–4 s before the stimulus (1–0 s before the visual cue) in the same condition in the left panels **(A,B,D)**. The results of Bonferroni test for the right GM amplitude in the auditory trial block are shown in panel **(C)** and those for the left TA in the tactile stimulus trial block are shown in panel **(E)**. N, trials with the cue predicting non-stimulation; F, trials with the cue predicting the stimulus to the forehead; B, trials with the cue predicting the stimulus to the back of the head; L, trials with the cue predicting the stimulus to the left side of the head; R, trials with the cue predicting the stimulus to the right side of the head. EMG, electromyography.

The EMG amplitude of the left GM in the auditory stimulus trial block is shown in Figure 5A. There was a significant interaction between the main effects [*F*_(16,208)_ = 1.847, *p* = 0.027]. The test of the simple main effect revealed a significant effect of time in the B condition [*F*_(4,260)_ = 3.498, *p* = 0.008]. The EMG amplitude at 3–0 s before the stimulus was significantly greater than that at 5–3 s before the stimulus in the B condition (*p* < 0.001). The test of the simple main effect did not reveal a significant effect of the stimulus in each time window.

The EMG amplitude of the right GM in the auditory stimulus trial block is shown in Figure 5B. There was a significant interaction between the main effects [*F*_(16,208)_ = 1.827, *p* = 0.029]. The test of the simple main effect revealed a significant effect of time in the L condition [*F*_(4,260)_ = 2.554, *p* = 0.039]. The EMG amplitude at 3–0 s before the stimulus was significantly greater than that at 5–3 s before the stimulus in the L condition (*p* < 0.05). The test of the simple main effect revealed a significant effect of the stimulus at 2–1 s [*F*_(4,260)_ = 2.764, *p* = 0.028] and 1–0 s [*F*_(4,260)_ = 2.731, *p* = 0.030] before the stimulus. As shown in Figure 5C, The EMG amplitude at 2–1 s before the stimulus in the B condition and that at 1–0 s before the stimulus in the L condition was greater than that in the N condition. The EMG amplitude at 2–0 s before the stimulus in the F condition was smaller than that in the N condition.

The EMG amplitude of the left TA in the tactile stimulus trial block is shown in Figure 5D. There was a significant interaction between the main effects [*F*_(16,208)_ = 1.901, *p* = 0.022]. The test of the simple main effect revealed a significant effect of time in the B [*F*_(4,260)_ = 3.405, *p* = 0.010], F [*F*_(4,260)_ = 3.278, *p* = 0.012], L [*F*_(4,260)_ = 5.854, *p* < 0.001], and R [*F*_(4,260)_ = 9.402, *p* < 0.001] conditions. The EMG amplitude at 5–4 s before the stimulus was significantly smaller than that at the other times, and that at 4–2 s before the stimulus was significantly smaller than that at 2–0 s before the stimulus in the B condition (*p* < 0.05). The EMG amplitude at 5–3 s before the stimulus was significantly smaller than that at 3–0 s before the stimulus (1–0 s before the visual cue) in the F condition (*p* < 0.05). The EMG amplitude at 5–4 s before the stimulus (1–0 s before the visual cue) was significantly smaller than that at 4–0 s before the stimulus, and that at 4–3 s before the stimulus was smaller than 3–2 and 1–0 s before the stimulus in the L condition (*p* < 0.05). The EMG amplitude at 5–4 s before the stimulus (1–0 s before the visual cue) was significantly smaller than that at 3–0 s before the stimulus, and that at 4–2 s before the stimulus was significantly smaller than 2–0 s before the stimulus in the R condition (*p* < 0.05). The test of the simple main effect revealed a significant effect of the stimulus at 2–1 s [*F*_(4,260)_ = 3.190, *p* = 0.014] and at 1–0 s [*F*_(4,260)_ = 3.001, *p* = 0.019] before the stimulus. As shown in Figure 5E, the EMG amplitude in the B, L, and R conditions at 2–1 s before stimulation was significantly greater than that in the N condition and the EMG amplitude at 1–0 s before stimulation in the F, B, L, and R conditions was significantly greater than that in the N condition.

#### Muscles without significant interaction between the main effects

**Figure 6:**
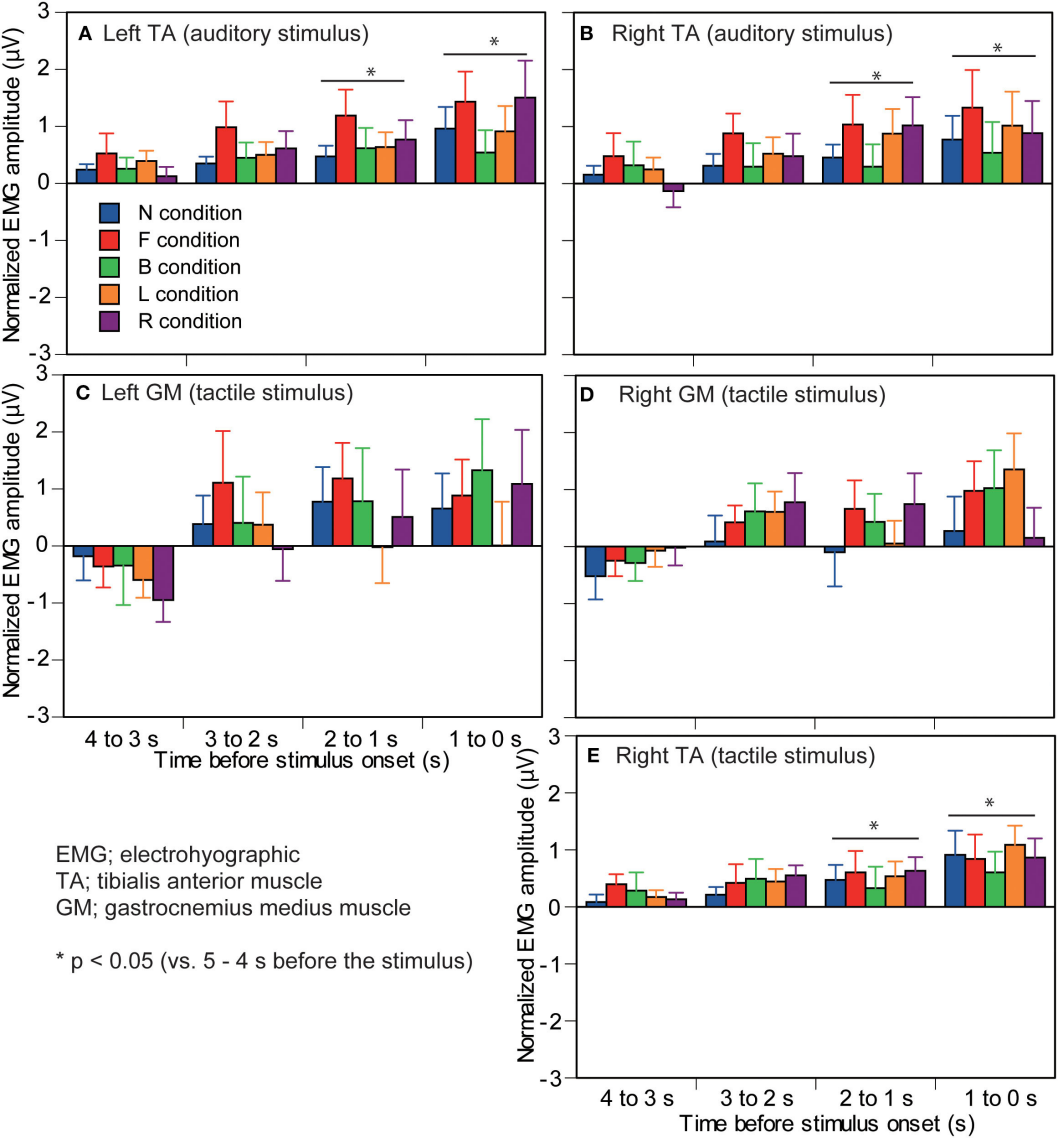
Effect of predicting the stimulus site on the EMG amplitude in which significant interaction between the main effects is not found. The left panels are the muscles in the left ankle **(A,C)** and the right panels are the muscles in the right ankle **(B,D,E)**. Upmost panels are the muscles in the auditory stimulus trial block **(A,B)**, and the other panels are the muscles in the tactile stimulus trial block **(C–E)**. The data are normalized by subtracting the average value at 1–0 s before the onset of the visual cue (5–4 s before the stimulus) from the values averaged at each 1 s in the time window between 4 and 0 s before the stimulus. Bars indicate the mean, and error bars indicate standard errors of the mean. Asterisks indicate significant differences from mean amplitude at the time 5–4 s before the stimulus (1–0 s before the visual cue) in the same condition. N, trials with the cue predicting non-stimulation; F, trials with the cue predicting the stimulus to the forehead; B, trials with the cue predicting the stimulus to the back of the head; L, trials with the cue predicting the stimulus to the left side of the head; R, trials with the cue predicting the stimulus to the right side of the head. EMG, electromyography.

The EMG amplitude of the left TA in the auditory stimulus trial block is shown in Figure 6A. There was no significant interaction between the main effects [*F*_(16,208)_ = 1.458, *p* = 0.118]. There was a significant main effect of time [*F*_(1.528,19.861)_ = 5.689, *p* = 0.016]. The EMG amplitude at 5–4 s before the stimulus (1–0 s before the visual cue) was significantly smaller than that at 2–0 s before the stimulus. The EMG amplitude at 1–0 s before the stimulus was significantly greater than that at 4–3 s before the stimulus. There was no significant main effect of the stimulus [*F*_(2.221,28.874)_ = 3.146, *p* = 0.053].

The EMG amplitude of the right TA in the auditory stimulus trial block is shown in Figure 6B. There was no significant interaction between the main effects [*F*_(16,208)_ = 0.695, *p* = 0.798]. There was a significant main effect of time [*F*_(1.402,18.226)_ = 5.384, *p* = 0.023]. The EMG amplitude at 5–4 s before the stimulus was significantly smaller than that at 2–0 s before the stimulus (*p* < 0.05). The EMG amplitude at 4–3 s before the stimulus was significantly smaller than that at 1–0 s before the stimulus (*p* < 0.05). There was no significant main effect of the stimulus [*F*_(2.045,26.579)_ = 1.111, *p* = 0.345].

The EMG amplitude of the left GM in the tactile stimulus trial block is shown in Figure 6C. There was no significant interaction between the main effects [*F*_(16,208)_ = 0.625, *p* = 0.861]. There was a significant main effect of time [*F*_(4,52)_ = 4.109, *p* = 0.006]. The EMG amplitude at 4–3 s before the stimulus was significantly smaller than that at 2–0 s before the stimulus (*p* < 0.05). There was no significant main effect of the stimulus [*F*_(4,52)_ = 1.447, *p* = 0.232].

The EMG amplitude of the right GM in the tactile stimulus trial block is shown in Figure 6D. There was no significant interaction between the main effects [*F*_(16,208)_ = 1.026, *p* = 0.431]. There was neither a significant main effect of the stimulus [*F*_(1.833,23.835)_ = 1.641, *p* = 0.216] nor of time [*F*_(2.173,28.254)_ = 3.043, *p* = 0.060].

The EMG amplitude of the right TA in the tactile stimulus trial block is shown in Figure 6E. There was no significant interaction between the main effects [*F*_(16,208)_ = 0.455, *p* = 0.965]. There was a significant main effect of time [*F*_(1.685,21.907)_ = 9.170, *p* = 0.002]. The EMG amplitude at 5–4 s before the stimulus was significantly smaller than that at 2–0 s before the stimulus (*p* < 0.05). The EMG amplitude at 4–3 s before the stimulus was significantly smaller than that at 1–0 s before the stimulus (*p* < 0.05). There was no significant main effect of the stimulus [*F*_(4,52)_ = 0.826, *p* = 0.515].

## Discussion

### Predicting auditory stimulus and body sway

There are defense mechanisms that keep the body away from the stimulus (Graziano and Cooke, 2006). The withdrawal reflex elicited by a noxious tactile stimulus and the startle response induced by the loud auditory stimulus are typical defense responses in humans (Sherrington, 1910; Landis and Hunt, 1939; Schouenborg et al., 1995; Clarke and Harris, 2004). Such defense mechanisms are activated even when tactile stimulation is given to the head; when tactile stimulation (air puff) was given to the head, monkeys moved their heads away from the stimulus (Cooke et al., 2003). Based on these findings, we hypothesized that the defense mechanisms may even be activated by predicting the forthcoming auditory stimulus site of the head in a quiet stance (Hypothesis 1). In this study, a visual cue predicting the auditory stimulus site induced a body sway in a direction contrary to the predicted stimulus site. This finding is in line with our Hypothesis 1 that predicting the auditory stimulus site of the head induces a body sway in the direction contrary to the predicted stimulus site to keep the head away from the stimulus and that this is mediated by the defense mechanism.

One alternative mechanism underlying the body sway induced by the prediction of the auditory stimulus site in a quiet stance is sound localization. The head was found to move to localize the sound direction (Wallach, 1939; Toyoda et al., 2011; Nojima et al., 2013; McAnally and Martin, 2014). Head movement reduced the front–back error of sound localization (Perrett and Noble, 1997; Iwaya et al., 2003). Based on these findings, one may speculate that the body sway induced by the prediction of the auditory stimulus site is partially explained by the head moving to improve sound localization. However, this view is unlikely to be true because keeping the head away from the auditory stimulus site does not seem to improve sound localization.

### Asymmetrical body sway

One interesting finding regarding the effect of the prediction of the auditory stimulus on the body sway was that the body sway induced by the prediction of the auditory stimulus given to the left side of the head tended to be much greater than that induced by the prediction of the stimulus given to the right side of the head. Moreover, the COP position significantly deviated rightward by predicting the left head stimulus immediately before the stimulus compared with the COP position immediately before the visual cue, but a significant deviation of the COP position over time was absent when predicting the right head stimulus. Those findings may be explained by an approach-avoidance associative network; the left hemisphere is related to the approach-related thought and the right hemisphere is related to the avoidance-related thought (Fetterman et al., 2013). A monaural auditory stimulus in one ear was found to increase the regional cerebral blood flow in the primary auditory area contralateral to the auditory stimulus side (Hirano et al., 1997); the auditory-evoked magnetic field was larger in the auditory cortex contralateral to the auditory stimulus side (Pantev et al., 1986); and a monaural auditory stimulus predominantly activated the contralateral auditory centers (Scheffler et al., 1998; Schönwiesner et al., 2007). This means that auditory stimulus to the left ear causes greater auditory input to the right hemisphere. Accordingly, the different effects of the prediction of the left auditory stimulus and that of the right auditory stimulus may be explained by a view that the body sways to keep the head away from the auditory stimulus site when the individual predicts auditory stimulus to the left side of the head because avoidance-related thought is processed in the right hemisphere, which receives its auditory input from the left ear.

### Predicting auditory stimulus-induced muscle activity

There was a significant simple main effect of the stimulus site on the EMG amplitude in the right GM. This means that the time course of the change in the right GM activity is dependent on the prediction of the stimulus site of the head. This predicted site-dependent activity of the right GM was present immediately before the stimulus; predicting that the stimulus to the back of the head significantly increased the activity of the right GM, and that to the forehead significantly decreased the activity of the right GM at 2–0 s before the stimulus. The time window of the predicted site-dependent change in the activity of the right GM was the same as the time window of the predicted site-dependent change in body sway (i.e., at 2–0 s before the stimulus). These findings indicate that the activity of the right GM is related to the body sway induced by the prediction of the auditory stimulus site.

The GM is activated eccentrically when the stance leg leans forward in the late stance phase of gait (Perry, 1992). This means that the GM in the stance leg contracts eccentrically when the lower leg leans forward. Accordingly, the increase in the activity of the right GM and forward body sway induced by predicting the auditory stimulus to the back of the head and the decrease in the activity of the right GM and backward body sway induced by the prediction of the forward head fits well with the kinesiological mechanism, that is, eccentric contraction of the ankle extensor (i.e., GM) to support the body against the forward body sway and vice versa. Thus, the right GM is likely to be a key muscle supporting the forward-leaned body induced by the prediction of the auditory stimulus to the back of the head.

On the one hand, the EMG amplitude in the right GM was significantly increased with the prediction of the auditory stimulus to the left head, and a similar increased tendency was induced by the prediction of the auditory stimulus to the right head. On the other hand, the EMG amplitude in the right GM was significantly increased by predicting the auditory stimulus to the back of the head but was significantly decreased by predicting the auditory stimulus to the forehead. That is, the direction of the change in the right GM activity was dependent on the predicted site of the stimulus in the sagittal plane. The ankle mainly contributes to the control of the anterior–posterior body sway in a quiet stance, although the hip mainly contributes to the control of the medial–lateral body sway (Winter, 1993). Accordingly, the different directions of the change in the right GM activity induced by the prediction of the stimulus to the forehead and that induced by that to the back of the head likely reflect ankle control of the anterior–posterior body sway in a quiet stance. Thus, Hypothesis 2 was supported for the prediction of the auditory stimulus.

In the present study, the effect of predicting the auditory or tactile stimulus on GM activity was asymmetrical. The change in the right GM activity was dependent on the predicted site of the auditory stimulus, but the left GM activity was not. All the participants were right-footed. Thus, the finding may be explained by the view that the prediction of the auditory stimulus site is associated with the activity in the ankle extensor of the dominant leg. Nevertheless, this view must be handled with caution, because this view is not in line with the previous findings on the contribution of the dominant and non-dominant leg to the control of the postural task. The contribution of the leg to the postural control has been found to not be different between the dominant and non-dominant leg sides (Paillard and No, 2020; Schorderet et al., 2021).

### Predicting tactile stimulus

A visual cue predicting the tactile stimulation site did not induce a body sway in the quiet stance. This finding was inconsistent with the finding on the prediction of the auditory stimulus site. In addition, the present finding on the prediction of the tactile stimulation site was not in line with a finding that tactile stimulus to the head activated the defense mechanism (Cooke et al., 2003). One explanation for the absence of an effect of predicting tactile stimulation on body sway is that the defense mechanism is not activated by the prediction of tactile stimulus to the head in a quiet stance.

In the present study, the order of the trial block was constant across the participants; the auditory stimulus trial block was first and the tactile stimulus trial block was second. Thus, the tactile stimulus trial block was preceded by the repetitive auditory stimuli. Habituation of the response occurs after the repetitive stimuli (Webster, 1971; Dimitrijevi et al., 1972). Thus, one may speculate that habituation induced by the repetitive auditory stimulus occurred in the second trial block (tactile stimulus trial block), and this may be a reason that the prediction of the tactile stimulus was not effective on the postural sway in quiet stance. However, in our opinion, this view is unlikely, because the interval between the trial blocks (20 min) was long enough to eliminate habituation. Moreover, because the stimulus modality was different between the two trial blocks, habituation induced by the repetitive auditory stimulation preceding the tactile stimulus trial block is likely minor.

There was a significant interaction between the main effects on the EMG amplitude in the left TA. The EMG amplitude increased immediately before the stimulus when the participants predicted the tactile stimulus to the head no matter where the predicted stimulus site was. Even though the amount of the increase was significantly different between the stimulus sites, the direction of the change was the same across all the stimulus conditions. Thus, this increase is likely due to predicting the presence of the stimulus no matter where the stimulus is given. In the right TA, the activity level increased immediately before the stimulus (1 - 0 s before the stimulus) across all the stimulus conditions. This means that the activity level of the TA increases immediately before the stimulus no matter whether the stimulation is given or not. Taken together, the increase in the TA activity is not related to the defense mechanism, in which the direction of the change in the EMG activity must be dependent on the predicted stimulus site.

### Amount of body sway

A static sound cue was found to decrease the amount of body sway in a stance (Gandemer et al., 2017). A rotating sound cue was also found to decrease the amount of body sway in a stance (Gandemer et al., 2014). Auditory stimulus reduced the amount of body sway in a stance (Agaeva and Altman, 2005; Ross and Balasubramaniam, 2015; Ross et al., 2016). When humans maintained their stance on an elevated ground surface, the COP displacement decreased, indicating that postural threat decreases body sway in a quiet stance (Carpenter et al., 2001; Brown et al., 2006). These previous findings are explained by the view that emotional stress decreases the amount of body sway in a stance. Predicting stimulus to the head likely induces emotional stress. This stress may raise the individual's alertness and influences the amount of body sway. According to these previous findings, we hypothesized that predicting the stimulus to the head decreases the amount of body sway (Hypothesis 3). However, this hypothesis was not supported; any significant change in the standard deviation of the COP was not revealed immediately before the stimulus.

The amount of the body sway (i.e., the SD of the COP) decreased immediately after the presentation of the visual cue (i.e., 4–3 s before the stimulus) compared with the time at 5–4 and/or 3–0 s before the stimulus. A visual cue was given at 4 s before the stimulus. Vision significantly contributes to head stabilization (Guitton et al., 1986). Thus, the finding is likely explained by the view that the freezing of the body to stabilize the head for viewing the visual cue lasted for 1 s immediately after the visual cue.

## Limitations

The experiment was conducted only for male participants. Thus, we could not compare the gender difference in the present study. Further studies are needed on female participants. One may raise a question of why the effect of stimulus modality was not examined in the present study. To test the effect of the stimulus modality, a large sample size is required for three-way ANOVA. In the present study, the sample size was 14. Accordingly, the effect of the stimulus modality was not tested, because the sample size was not enough to conduct three-way ANOVA. These are the limitations of the present study.

## Conclusion

A visual cue predicting an auditory stimulus to the back of the head induced a forward body sway, while the visual cue predicting an auditory stimulus to the forehead induced a backward body sway. The cue predicting the auditory stimulus to the left side of the head induced a rightward body sway, while the cue predicting the auditory stimulus to the right side of the head induced a leftward body sway. These findings support our hypothesis that predicting the auditory stimulus site of the head induces a body sway in the direction contrary to the predicted stimulus site to keep the head away from the stimulus, mediated by the defense mechanism. The right gastrocnemius muscle contributes to the control of the body sway in the anterior–posterior axis related to this defense mechanism.

## Data availability statement

The original contributions presented in the study are included in the article/Supplementary material, further inquiries can be directed to the corresponding authors.

## Ethics statement

The studies involving human participants were reviewed and approved by Ethics Committee of Osaka Prefecture University. The patients/participants provided their written informed consent to participate in this study.

## Author contributions

NH and KH: study design, conducting experiment, data analysis, and writing manuscript. HK, MM, and HO: conducting experiment. All authors contributed to the article and approved the submitted version.
